# Analysis of signature genes and association with immune cells infiltration in pediatric septic shock

**DOI:** 10.3389/fimmu.2022.1056750

**Published:** 2022-11-10

**Authors:** Jiajie Fan, Shanshan Shi, Yunxiang Qiu, Mingnan Liu, Qiang Shu

**Affiliations:** ^1^ Department of Cardiac Intensive Care Unit, The Children’s Hospital, Zhejiang University School of Medicine, National Clinical Research Center for Child Health, Hangzhou, China; ^2^ Department of Cardiac Surgery, The Children’s Hospital, Zhejiang University School of Medicine, National Clinical Research Center for Child Health, Hangzhou, China

**Keywords:** children, septic shock, weighted gene co-expression network analysis, signature gene, immune cells infiltration

## Abstract

**Background:**

Early diagnosis of septic shock in children is critical for prognosis. This study committed to investigate the signature genes and their connection with immune cells in pediatric septic shock.

**Methods:**

We screened a dataset of children with septic shock from the GEO database and analyzed differentially expressed genes (DEGs). Functional enrichment analysis was performed for these DEGs. Weighted gene co-expression network analysis (WCGNA) was used to screen the key modules. Least absolute shrinkage and selection operator (LASSO) and random forest analysis were finally applied to identify the signature genes. Then gene set enrichment analysis (GSEA) was exerted to explore the signaling pathways related to the hub genes. And the immune cells infiltration was subsequently classified *via* using CIBERSORT.

**Results:**

A total of 534 DEGs were screened from GSE26440. The data then was clustered into 17 modules *via* WGCNA, which MEgrey module was significantly related to pediatric septic shock (cor=−0.62, *p*<0.0001). LASSO and random forest algorithms were applied to select the signature genes, containing UPP1, S100A9, KIF1B, S100A12, SLC26A8. The receiver operating characteristic curve (ROC) of these signature genes was 0.965, 0.977, 0.984, 0.991 and 0.989, respectively, which were verified in the external dataset from GSE13904. GSEA analysis showed these signature genes involve in positively correlated fructose and mannose metabolism and starch and sucrose metabolism signaling pathway. CIBERSORT suggested these signature genes may participate in immune cells infiltration.

**Conclusion:**

UPP1, S100A9, KIF1B, S100A12, SLC26A8 emerge remarkable diagnostic performance in pediatric septic shock and involved in immune cells infiltration.

## Introduction

Sepsis is a life-threatening generalized inflammatory response primarily caused by a disordered defense against pathogen infection, usually along with abnormal immune response as well as multiple organ dysfunction (1). As noted by the World Health Organization (WHO), sepsis is now becoming a major worldwide public health problem, especially among children (2). Globally, there are an estimated 1.2 million cases of sepsis in children each year (3). The mortality rate for pediatric sepsis is approximately 1% to 25% (4, 5).

Septic shock, the most serious complication of sepsis, is defined as patients accompanied by acute circulatory failure, which is characterized by persistent hypotension even with adequate volumetric resuscitation, and is otherwise unexplained (6). The mortality rate for pediatric septic shock was as high as 50% in the 1980s (7, 8). Fortunately, in the past few decades, with the development of diagnosis and treatment, the mortality rate of children with septic shock has decreased in developed nations (9). However, the mortality rates in third world countries are still extremely high as before (10, 11).

Clinically, the first few hours after the diagnosis of pediatric septic shock are known as the “gold time” for survival, when targeted treatment is urgently needed to carry out as soon as possible (12, 13). It has been reported that without proper resuscitation and restoration of blood pressure, the clinical condition of children can deteriorate rapidly and the risk of death increases by 40% per hour (14, 15). Treatment of sepsis or septic shock should include treatment of infection and source control, reversal of hemodynamic abnormalities, and preservation of end-organ perfusion. The key to the treatment of septic shock is the control of infection, the reversal of hemodynamic abnormalities, the guarantee of the perfusion of important organs, and stabilization of airway and adequate breathing with oxygen supply (16). Thus, early diagnosis as well as timely and effective intervention is crucial to improve prognosis and reduce mortality of children with septic shock.

The role of innate and adaptive immunity in the defense against pathogens is irreplaceable. However, sepsis can induce immune resistance of the host, so that the invading pathogens cannot be eliminated immediately, which make the host more prone to refractory infection or new secondary infection (17). According to a small sample study, pediatric septic shock showed early adaptive immune suppression and developed subsequent infectious complications when contrast to hygeian peers (18). Therefore, further exploration of the underlying mechanism of immune suppression in septic shock emerge a favorable prospect in the treatment of pediatric septic shock.

At the present article, we employed multiple bioinformatic approaches to pick up signature genes in pediatric septic shock. These genes displayed remarkable diagnostic performance and were validated in external dataset. Finally, we further evaluated the enrichment signaling pathways of these genes and their roles in the immune cell infiltration, in order to provide new insights for the diagnosis and treatment of clinicians.

## Method and material

### Data sources

For the current study, two datasets, namely GSE26440 and GSE13904 (Platforms: GPL570[HG-U133_Plus_2] Affymetrix Human Genome U133 Plus 2.0 Array), have been downloaded from Gene Expression Omnibus (GEO) (http://www.ncbi.nlm.nih.gov/geo/). As a training set, GSE26440 contained 130 patients, including 98 children with septic shock and 32 normal controls, while a validation group consisting of 106 children with septic shock and 18 normal controls was obtained from GSE13904.

### Identification of DEGs

Using R software’s limma package (19), differentially expressed genes (DEGs) between septic shock cohort and control cohort were analyzed, with the following criterion: adjust *p* value <0.05 and |log fold change (FC)| > 1. The volcano plot was generated to show these DEGs, while the top 50 up-regulated and the top 50 down-regulated DEGs were displayed by the heatmap.

### Functional and pathway enrichment analyses

The clusterProfiler package in R was used for functional enrichment analyses of DEGs based on Gene Ontology (GO) and Kyoto Encyclopedia of Genes and Genomes (KEGG) (20). As part of the GO analysis, three categories were identified, biological process (BP), cellular component (CC), and molecular function (MF), which was contributed to explore the biological processes of these DEGs. Potential signaling pathways were explored using KEGG analysis.

### Weighted gene co-expression network analysis

Based on the scale-free topology criterion, the co-expression network in the GSE26440 cohort was constructed by weighted gene co-expression network analysis (WGCNA) (21). The pickSoftThreshold function of the WGCNA package was performed to calculate the soft threshold power as well as adjacencies. The adjacency matrix was then converted into a topological overlap matrix (TOM) and the corresponding dissimilarity was calculated to perform hierarchical clustering analysis. The dynamic tree cutting method with a minimum module size of 50 were used to identify the co-expressed gene modules. We then measured the connection between the gene modules and children with sepsis shock *via* gene significance (GS) values as well as module membership (MM) values and ultimately identified the key modules.

### Signature gene identification

We identified candidate hub genes by the intersection of DGEs and key module genes. Subsequently, two machine learning algorithms, namely least absolute shrinkage and selection operator (LASSO) as well as random forest were exerted to screen hub genes.

LASSO analysis was implemented using the glmnet package with penalty parameters for 10-fold cross-validation, which is superior to regression analysis method in evaluating high-dimensional data (22). Moreover, we applied the R package “randomforest” to classify the DEGs for hub genes. The random forest model determined the optimal number of variables by computing the average error rate of candidate hub genes (23). Then, we calculated the error rate for each of one to 500 trees and determined the optimal number of trees based on the lowest error rate. A random forest tree model was built when the above parameters were determined. Finally, the feature importance scores of each candidate hub gene were identified, and the genes with an importance value greater than 0.25 were selected. The intersection genes of these two machine learning algorithms were as the signature genes of children with sepsis shock. The area under curve (AUC) of the receiver operating characteristic curve (ROCs) was used to assess the diagnostic efficiency of these signature genes. An AUC greater than 0.7 indicated favorable diagnostic performance.

### Gene set enrichment analysis

To identify the association between the of signature genes and signaling pathways, we grouped the septic shock cohort according to the median value of hub gene expression, and performed gene set enrichment analysis (GSEA) on different subgroups with adjusted *p*< 0.05 (24).

### Immune cell infiltration

The CIBERSORT, a method using the principle of linear support vector regression to deconvolute the expression matrix of 22 human immune cell subtypes, was used to explore the discrepancy in immune cell between children with sepsis shock and healthy subjects (25). Subsequently, we screened out the immune cells that showed significant differences in infiltration between pediatric septic shock and normal children, and analyzed the correlation with the signature genes by spearman method.

### Statistical analysis

**Figure 1:**
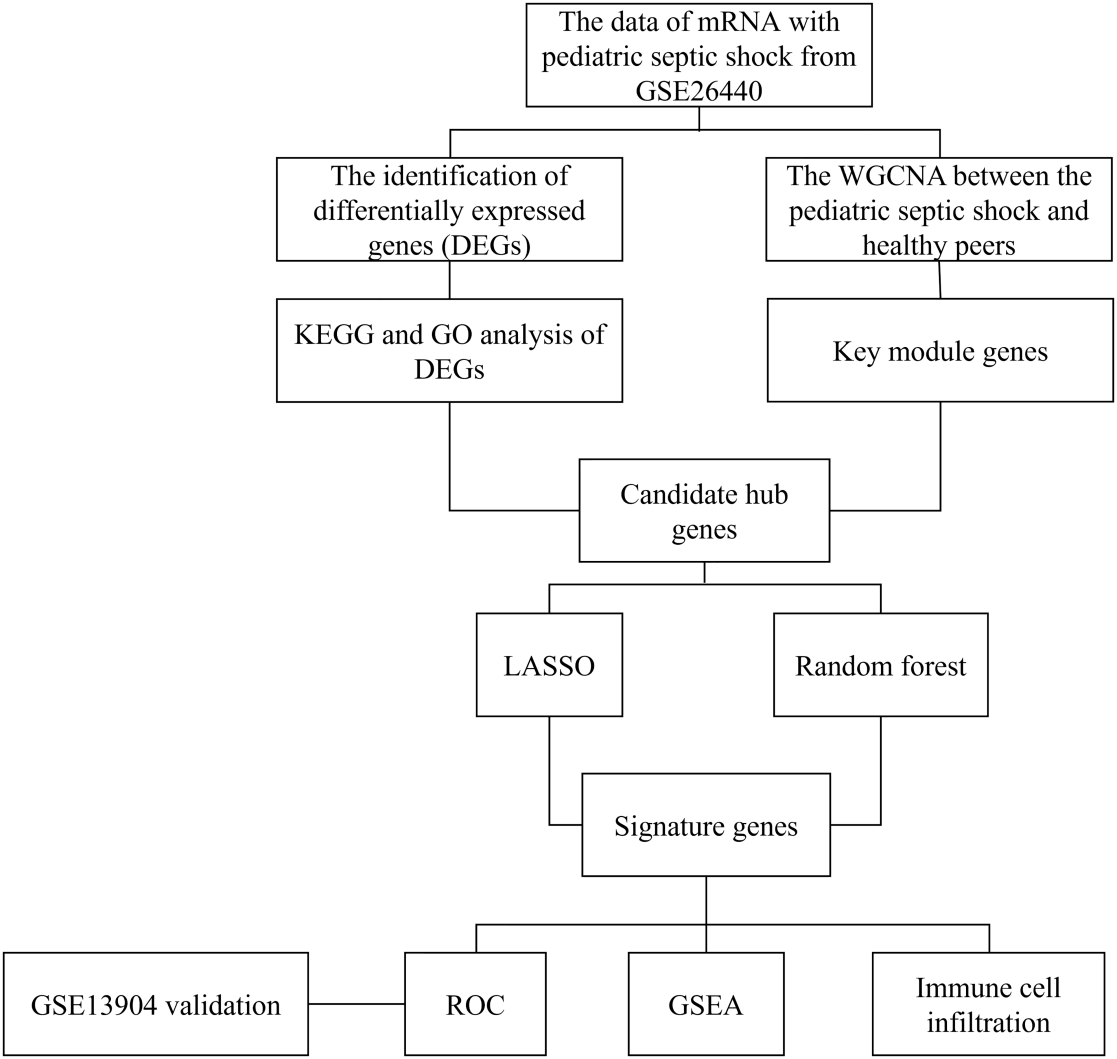
The flow chart of this research.

All statistical analyses in the present study were implemented using R software (version 4.1.2). Unless otherwise stated, *p*<0.05 was deemed as statistically significant, and all *p* values were two-tailed. The flow chart of this research was shown in **Figure 1**.

## Results

### Identification of DEGs between sepsis and control

**Figure 2:**
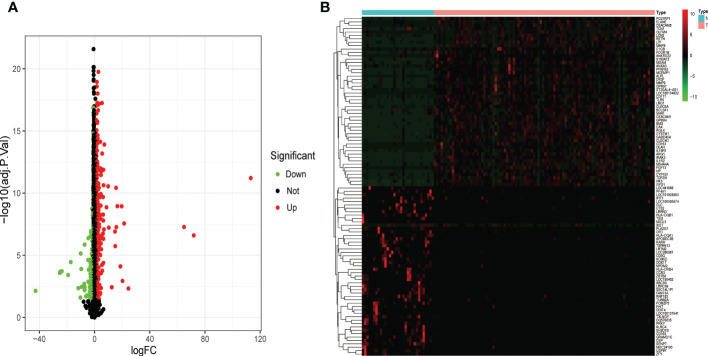
Identification of the DEGs in pediatric septic shock. **(A)** Volcano showed expression of DEGs between the pediatric septic shock and healthy cohort. **(B)** The heatmap showed the top 50 up-regulated DEGs and 50 down-regulated DEGs.

DEGs from children with septic shock and normal controls were analyzed using the “limma” package. A total of 534 DEGs were finally screened, of which 346 genes were up-regulated and 188 genes were down-regulated (**Figure 2A**). The heatmap showed the top 50 up-regulated and top 50 down-regulated DEGs between children with septic shock and healthy group (**Figure 2B**).

### Function enrichment analysis

**Figure 3:**
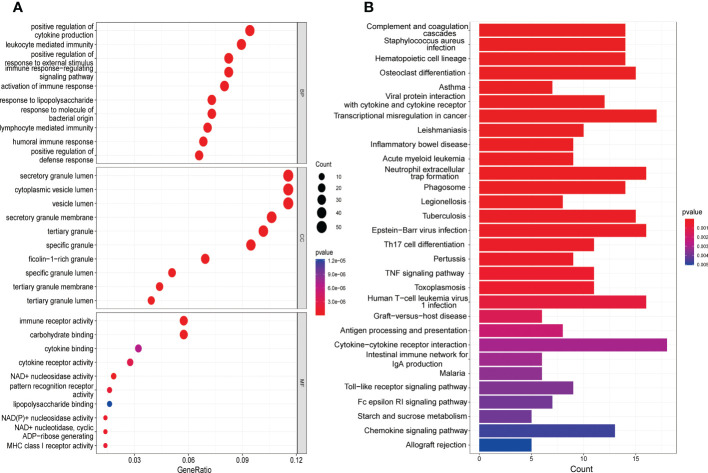
Functional enrichment analysis of DEGs. **(A)** The top 10 functional enrichment in BP, CC, and MF analysis, respectively. **(B)** The KEGG analysis of DEGs.

The GO analysis consists of three categories (**Figure 3A**), namely BP, CC, as well as MF. The BP analysis displayed that positive regulation of cytokine production, leukocyte mediated immunity and positive regulation of response to external stimulus were significantly enriched. In CC analysis, secretory granule lumen, cytoplasmic vesicle lumen and vesicle lumen occupied the top three positions. Moreover, immune receptor activity, carbohydrate binding as well as endopeptidase activity played an essential role in MF. As shown in the KEGG analysis, the top 3 enriched pathways were mainly complement and coagulation cascades, staphylococcus aureus infection as well as hematopoietic cell lineage (**Figure 3B**).

### Construction of the weighted gene co-expression network

**Figure 4:**
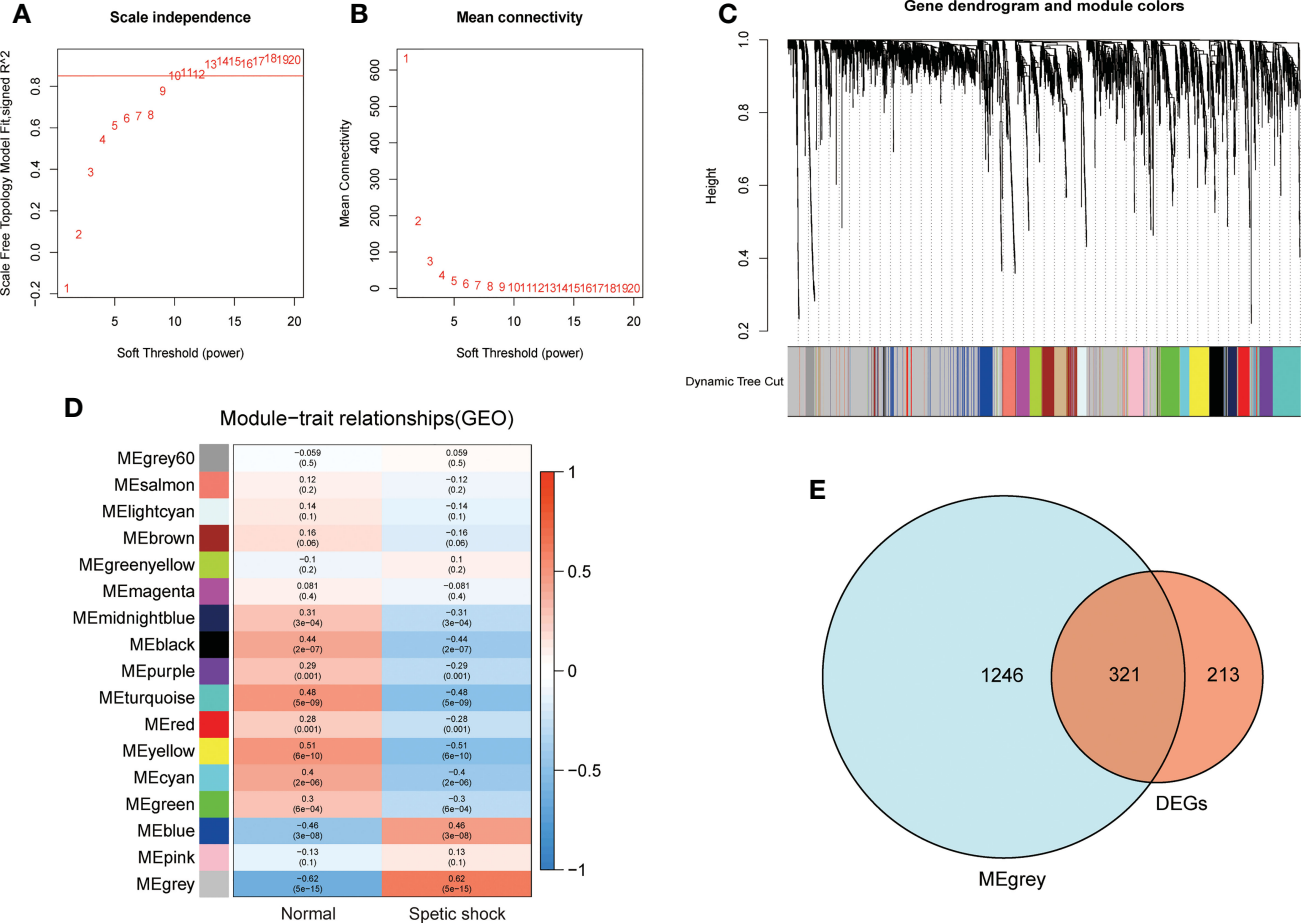
The WGCNA analysis of GSE26440 and identification of candidate hub genes. **(A)** The soft threshold power of WGCNA. **(B)** The mean connectivity of WGCNA. **(C)** The cluster dendrogram of WGCNA. **(D)** The clustered modules of WGCNA. **(E)** The veen plot showed the interaction between DEGs and genes in MEgrey module.

Children with septic shock and healthy subjects were analyzed using WGCNA package in R software, and a scale-free co-expression network was established, and the soft threshold power was determined as 10 with a scale-free index of 0.85 as well as a relatively favorable mean connectivity (**Figures 4A, B**). The cluster dendrogram was shown in **Figure 4C**. Finally, the data was clustered into 17 modules (**Figure 4D**). The correlation between each module and children with septic shock was calculated. The results indicated that the MEgrey module was significantly related to children with sepsis shock (cor=−0.62, *p*<0.0001). Herein, MEgrey module included 1567 genes, which was regarded as a pivotal module related to children with sepsis shock. The overlap between the DEGs and genes in MEgrey module was shown in **Figure 4E**.

### Selection of signature genes *via* LASSO and random forest algorithms

**Figure 5:**
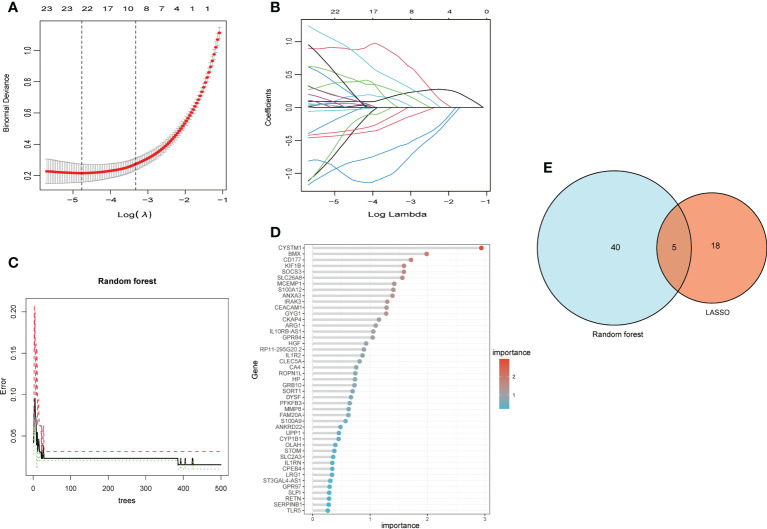
The machine algorithms for signature genes. **(A)** Penalty plot of the LASSO model with error bars denoting standard errors. **(B)** LASSO plot showed the variations in the size of coefficients for parameters shrank as the value of k penalty increased. **(C)** The error rate confidence intervals for random forest model. **(D)** The relative importance of genes is more than 0.25 in random forest model, **(E)** The interaction of the LASSO and random forest algorithms.

Two machine algorithms were applied to screen out signature genes from candidate key genes in children with septic shock. For the LASSO analysis selected 23 signature genes (**Figures 5A, B**), while in the random forest analysis, 45 signature genes were determined with relative importance more than 0.25 (**Figures 5C, D**). These screened out signature genes were displayed in **Table 1**. Five signature genes were finally determined *via* the interaction of these two algorithms, containing UPP1, S100A9, KIF1B, S100A12, SLC26A8 (**Figure 5E**).

**Table 1:** The key genes screened out through LASSO and random forest analysis.

LASSO analysis	Random forest algorithms
UPP1, S100A9, KIF1B, RP11-295G20.2, LMNB1, MGST1, S100A12, FLJ12120, GRINA, MAP3K7CL, NARF, CX3CR1, SLC26A8, CTSW, RPS4Y1, RNF182, HIST1H4H, MINOS1P1, IGJ, ASAP1-IT1, IL2RB, TGFB1I1, C4BPA	CYSTM1, BMX, CD177, KIF1B, SOCS3, SLC26A8, MCEMP1, S100A12, ANXA3, IRAK3, CEACAM1, GYG1, CKAP4, ARG1, IL10RB-AS1, GPR84, HGF, RP11-295G20.2, IL1R2, CLEC5A, CA4, ROPN1L, HP, GRB10, SORT1, DYSF, PFKFB3, MMP8, FAM20A, S100A9, ANKRD22, UPP1, CYP1B1, OLAH, STOM, SLC2A3, IL1RN, CPEB4, LRG1, ST3GAL4-AS1, GPR97, SLPI, RETN, SERPINB1, TLR5

### Diagnostic efficacy of signature genes in predicting septic shock

**Figure 6:**
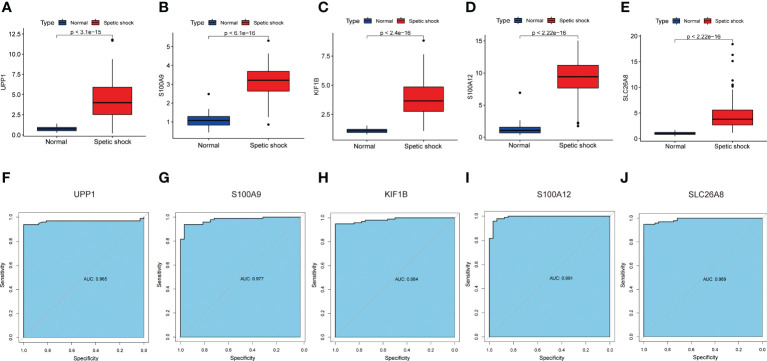
The performance of the signature genes in GSE26440. **(A–E)** The expression of signature genes between the pediatric septic shock and healthy cohort. **(F–J)** ROC showed the diagnostic performance of the signature genes.

**Figure 7:**
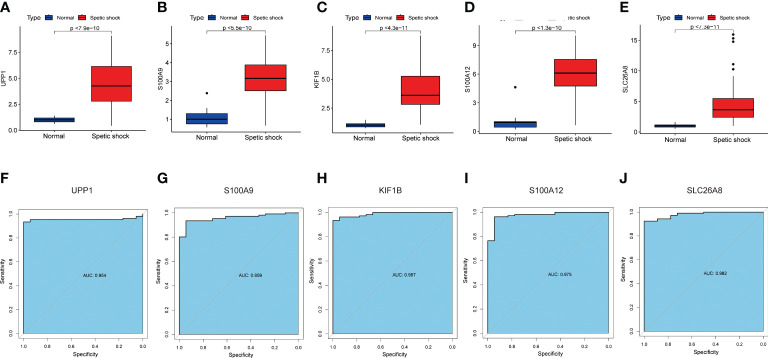
The performance of the signature genes in GSE13904. **(A–E)** The expression of signature genes between the pediatric septic shock and healthy cohort. **(F–J)** ROC showed the diagnostic performance of the signature genes.

The screened signature genes were highly expressed in children with sepsis shock than those in healthy children, suggesting that these genes may play a potential role in pediatric sepsis shock (**Figures 6A-E**). Furthermore, the area under curve (AUC) of the receiver operating characteristic curve (ROC) of these signature genes was 0.965 of UPP1, 0.977 of S100A9, 0.984 of KIF1B, 0.991 of S100A12, 0.989 of SLC26A8 respectively (**Figures 6F-J**). We also evaluated the diagnostic efficiency of each signature gene in predicting pediatric septic shock in an external validation cohort. Consistent with GSE26440, these signature genes were highly expressed in children with septic shock (**Figures 7A-E**). The AUC values of ROC were 0.954 of UPP1, 0.959 of S100A9, 0.987 of KIF1B, 0.975 of S100A12, 0.982 of SLC26A8, respectively (**Figures 7F-J**). These phenomena indicated that the screened signature genes had remarkable diagnostic efficiency in forecasting pediatric septic shock.

### GSEA analysis

**Figure 8:**
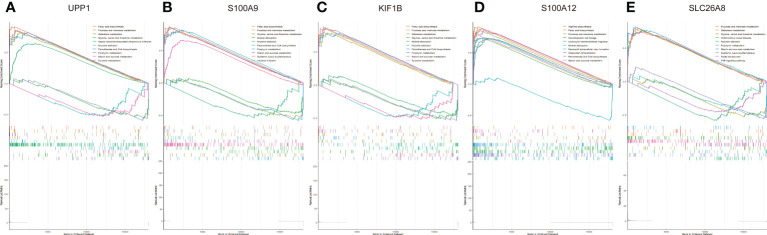
The GSEA of the signature genes in pediatric. **(A)** The GSEA of UPP1 in pediatric septic shock. **(B)** The GSEA of S100A9 in pediatric septic shock. **(C)** The GSEA of KIF1B in pediatric septic shock. **(D)** The GSEA of S100A12 in pediatric septic shock. **(E)** The GSEA of SLC26A8 in pediatric septic shock.

We assessed signaling pathways associated with signature genes *via* GSEA analysis. The top 10 signaling pathways were displayed in **Figure 8**. The results showed that UPP1 was significantly correlated with fatty acid biosynthesis, fructose and mannose metabolism, galactose metabolism, glycine, serine and threonine metabolism, Kaposi sarcoma-associated herpesvirus infection, nicotine addiction, pantothenate and CoA biosynthesis, porphyrin metabolism, starch and sucrose metabolism, tyrosine metabolism. The expression of S100A9 significantly correlated with fatty acid biosynthesis, fructose and mannose metabolism, galactose metabolism, glycine, serine and threonine metabolism, mineral absorption, nicotine addiction, pantothenate and CoA biosynthesis, porphyrin metabolism, starch and sucrose metabolism, systemic lupus erythematosus, yersinia infection. The expression of KIF1B significantly correlated with fatty acid biosynthesis, fructose and mannose metabolism, galactose metabolism, glycine, serine and threonine metabolism, mineral absorption, nicotine addiction, pantothenate and CoA biosynthesis, porphyrin metabolism, starch and sucrose metabolism, tyrosine metabolism. The expression of S100A12 significantly correlated with arginine biosynthesis, fatty acid biosynthesis, fructose and mannose metabolism, hematopoietic cell lineage, leukocyte transendothelial migration, mineral absorption, neutrophil extracellular trap formation, osteoclast differentiation, pantothenate and CoA biosynthesis, starch and sucrose metabolism. The expression of SLC26A8 significantly correlated with fructose and mannose metabolism, galactose metabolism, glycine, serine and threonine metabolism, inflammatory bowel disease, nicotine addiction, porphyrin metabolism, starch and sucrose metabolism, systemic lupus erythematosus, taste transduction, TNF signaling pathway. Taken together, these genes all positively correlated fructose and mannose metabolism signaling pathway as well as starch and sucrose metabolism signaling pathway.

### Immune cell infiltration

**Figure 9:**
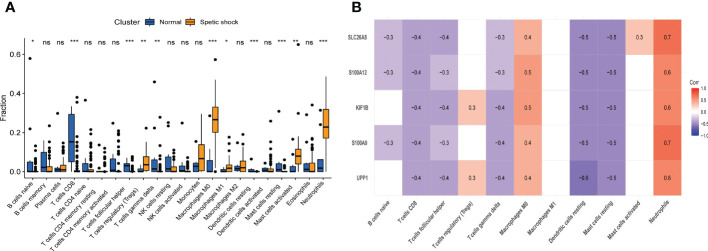
The immune cell infiltration association with signature genes. **(A)** The immune cell infiltration between the pediatric septic shock and healthy cohort. **(B)** The association between signature genes and significantly different immune cell infiltration. "ns" means P ≥ 0.01. *P < 0.01, **P < 0.001, and ***P < 0.0001.

Immunological features were evaluated according to immune cell infiltration. Compared with normal children, children with septic shock have higher T regulatory cells (Tregs), M0 macrophages, M1 macrophages, activated mast cells, neutrophils infiltration and lower naive B cells, CD8^+^ T cells, follicular helper T cells, gamma delta T cells, resting dendritic cells, resting mast cells infiltration (**Figure 9A**). All hub genes were negatively correlated with the infiltration of CD8^+^ T cells, follicular helper T cells, gamma delta T cells, resting dendritic cells, and resting mast cells, and positively correlated with the infiltration of M0 macrophages and neutrophils. S100A9, S100A12, SLC26A8 were negatively correlated with naive B cells, while UPP1, KIF1B were positively correlated with Tregs (**Figure 9B**). Only SLC26A8 was positively correlated with activated mast cells.

## Discussion

Pediatric septic shock is a highly lethal acute systemic inflammatory reaction with multiple organ dysfunction (13). Early diagnosis and treatment are critical to improving the prognosis of children with septic shock. More and more studies have shown that immune cell infiltration plays a non-negligible role in sepsis shock (26, 27). The present study assessed the DEGs between the pediatric sepsis shock and normal cohort and explored the key module based on WGCNA. The signature genes associated with pediatric septic shock was identified by LASSO analysis as well as random forest analysis, including UPP1, S100A9, KIF1B, S100A12, SLC26A8. We then verified these signature genes in external validation set. Subsequently, GSEA analysis was exerted to explore the signaling pathways related to hub genes. Finally, CIBERSORT algorithm was applied to analyze the immune cell infiltration between the pediatric sepsis shock and healthy groups the correlation with the signature genes.

Uridine phosphorylase 1(UPP1) encodes uridine phosphorylase, a key enzyme participates in the regulation of intracellular uridine homeostasis and the metabolism of pyrimidine ribonucleosides (28). Mike et al. reported that the expression of UPP1 was up-regulate in young sepsis rat when compared with aged sepsis rat (29). Consistent with our results, Guoli et al. also found that UPP1 was highly expressed in children with septic shock (30). These suggested that UPP1 may involve in young sepsis patients. Recently, UPP1 also has been reported to play a pivotal role in inflammatory and immune biological processes such as respiratory allergy (31) and chronic atrophic gastritis (32). Thus, UPP1 may affect the occurrence and progression of pediatric sepsis shock by regulating inflammatory and immune responses.

S100A9 is mainly derived from immune cells, such as macrophages and neutrophils, and is a member of the alarmins family, which functions in immune defense and homeostasis as well as mediating inflammatory responses (33, 34). It has been reported that patients with higher serum level of S100A9 are more susceptible to sepsis-related organ dysfunction (35). Mechanically, the S100A8/S100A9 complex can act as an endogenous activator of toll-like receptor 4 (TLR4), thereby enhancing phagocytic activation upstream of tumor necrosis factor α (TNF-α)-dependent action and promoting lethality in septic shock (36). Endotoxin tolerance was an essential immune dysfunction associated with sepsis (37). The expression of S100A9 was significantly elevated in the *in vitro* model of endotoxin tolerance, suggesting that S100A9 may be a novel biomarker of endotoxin tolerance and providing valuable information for immunotherapy in patients with sepsis shock (38). Furthermore, blockade of S100A9 can reduce the infiltration and activation of neutrophil and prevent sepsis-related pulmonary edema and tissue damage, suggesting that S100A9 may be a key target for alleviating sepsis-related injury (39).

The kinase protein family member 1B (KIF1B) gene belongs to the kinase protein superfamily and are mainly engaged in the intracellular transport of mitochondrial as well as synaptic vesicle precursors (40). KIF1B cooperates with the mitochondrial metalloproteinase YME1 like 1 ATPase (YME1L1) to mediate the fission and apoptosis of mitochondrial (41). In the early stage of sepsis, mitochondrial respiration is directly blocked by accumulated nitric oxide, which resulting in body shock (42). The underlying role of KIF1B in mitochondria implied that KIF1B may be critical in septic shock.

S100A12 is a calcium-binding protein expressed in the cytoplasm of neutrophils and also found in monocytes and lymphocytes (43, 44).The plasma level of S100A12 in patients with septic shock have been reported to be higher than in healthy volunteers (45). Notably, S100A12 has been regarded as a biomarker of the activating neutrophil in inflammatory diseases (46). S100A12 can provoke pro-inflammatory responses *via* binding to advanced glycation end products (RAGE) (47). Besides, as an endogenous TLR4 ligand, S100A12 can induce the activation of monocyte, thereby exerting an amplifying role in innate immunity during early inflammation and septic progression (48).

SLC26A8, also known as testis anion transporter 1 (TAT1), is identified as a novel member of the SLC26 family. At present, there are few reports on the function of SLC26A8. TAT1 is thought to be specifically expressed in spermatocytes and spermatids and interact with MgcRacGAP in these cells (49), which displays moderate transport of oxalate, Cl^−^ and SO42^−^ (50). However, the role of SLC26A8 in pediatric septic shock remains unclear.

Carbohydrates, the most abundant molecules in our daily life, are classified into three major categories according to their structure: simple sugars, such as glucose or sucrose; complex carbohydrates, such as starch; glycoconjugates, which are covalently modified forms of glucose that bind to proteins or lipids (51). Notably, the carbohydrates metabolism has been reported to involve in multiple pathological process, including inflammation, tumorigenesis as well as immune response (52–54).The present study indicated that the fructose and mannose metabolism signaling pathway as well as starch and sucrose metabolism signaling pathway participated in the children with septic shock. These results suggested that the carbohydrates metabolism may play a key role in septic shock. Moreover, it has been reported that fructose can reprograms glutaminolysis and oxidative metabolism to underpins lipopolysaccharide (LPS)-induced inflammation (55). It was well known that LPS, also known as endotoxin, was regarded as a pivotal molecule in the pathogenesis of septic shock (56).

Studies have shown that hyperinflammatory responses in septic shock often coexist with immunosuppression, resulting in undesirable treatment and poor prognosis (27, 57). Recently, study have reported that Tregs may act as an essential component of the immune dysfunction in sepsis (58). Monneret et al. found a higher level of circulating Tregs in patients with septic shock (59). Mechanically, circulating Tregs induced immunosuppression in septic shock patients by expressing tumor necrosis factor receptor type 2 (TNFR2) (60). Macrophages comprised a heterogeneous population of cells that can differentiate into different subtypes (61). Infection-induced acute inflammatory immune response lead to the transformation of M0 macrophages into M1 type, and these M1 macrophages can secrete a large number of pro-inflammatory molecules, including chemokines, cytokines, and reactive oxygen species (ROS), which severed as key factors in the progression of sepsis (62). Consistent with this phenomenon, mast cells also secreted multifunctional cytokines, nitric oxide (NO) and oxidants, which involved in septic shock (63). During septic shock, neutrophils were de-interacted with other immune cells and involved in the inflammatory response (64). Otherwise, neutrophils also mediated adaptive immune suppression *via* lymphocyte depletion in patients with septic shock (65). Although B cells were indispensable to the immune response to infection, patients with septic shock showed significantly fewer circulating B cells and prone to a depletion-like regulatory profile (66). Therefore, further studies are urgently needed to evaluate the role of B lymphocytes in sepsis-induced immunosuppression.

With a better understanding of how sepsis modulates the host immune response, new therapeutic agents, especially immune-based ones, are being developed. More specifically, IL-7, IL-15, granulocyte-macrophage colony stimulating factor (GM-CSF), anti-programmed cell death receptor-1 (PD-1), and anti- B- and T-lymphocyte attenuator (BTLA) targeted the immunosuppressive status of critically patients with sepsis shock (67). In particular, PD-1 specific antibodies and recombinant IL-7 can reverse the basic immune deficiency of sepsis, improve the survival rate of a variety of clinically relevant septic animal models, and show good clinical tolerance (17). Immunotherapy is promising for sepsis, however, the use of these cytokines and co-inhibitory molecules in sepsis, especially in children with septic shock, still needs to be further verified.

Our study also has certain limitations. First, the data for this study obtained from public databases and involved small samples, which may result in selected bias. However, the external validation database confirmed the reliability of our analysis. Second, molecular experiments as well as follow-up of larger clinical samples are required to further validate the results. Third, further studies are needed to confirm whether immune cell infiltration in hub genes is associated with the progression of pediatric septic shock.

## Conclusion

In summary, the present study screened out five signature genes, namely UPP1, S100A9, KIF1B, S100A12, SLC26A8, which showed prominent value in early diagnosis of pediatric septic shock. Besides, we also explored the immune cell infiltration in children with septic shock and their correlation with signature gene, which provided a new perspective for the role of immunity in pediatric septic shock.

## Data availability statement

The original contributions presented in the study are included in the article/supplementary material. Further inquiries can be directed to the corresponding author.

## Author contributions

JF and QS designed the study and drafted the manuscript. SS and YQ analyzed data and typeset figures. JF and ML revised the manuscript. All authors contributed to the article and approved the submitted version.

## Acknowledgments

The authors also thank the GEO database for the open access to the data and the reviewers for their constructive comments.

## Conflict of interest

The authors declare that the research was conducted in the absence of any commercial or financial relationships that could be construed as a potential conflict of interest.

## Publisher’s note

All claims expressed in this article are solely those of the authors and do not necessarily represent those of their affiliated organizations, or those of the publisher, the editors and the reviewers. Any product that may be evaluated in this article, or claim that may be made by its manufacturer, is not guaranteed or endorsed by the publisher.

## Abbreviations

WHO, World Health Organization; GEO, Gene Expression Omnibus; DEGs, differentially expressed genes; GO, Gene Ontology; KEGG, Kyoto Encyclopedia of Genes and Genomes; BP, biological process; CC, cellular component; MF, molecular function; WGCNA, weighted gene co-expression network analysis; TOM, topological overlap matrix; GS, gene significance; MM, module membership; LASSO, least absolute shrinkage and selection operator; GSEA, gene set enrichment analysis; AUC, area under curve; ROC, receiver operating characteristic curve; Tregs, regulatory T cells; UPP1, uridine phosphorylase 1; TLR4, toll-like receptor 4; TNF-α, tumor necrosis factor α; KIF1B, kinase protein family member 1B; YME1L1, YME1 like 1 ATPase; RAGE, advanced glycation end products; TAT1, testis anion transporter 1; LPS, lipopolysaccharide; TNFR2, tumor necrosis factor receptor type 2; ROS, reactive oxygen species; NO, nitric oxide.
