# Corrigendum: Analysis of signature genes and association with immune cells infiltration in pediatric septic shock

**DOI:** 10.3389/fimmu.2023.1241047

**Published:** 2023-07-17

**Authors:** Jiajie Fan, Shanshan Shi, Yunxiang Qiu, Mingnan Liu, Qiang Shu

**Affiliations:** ^1^ Department of Cardiac Intensive Care Unit, The Children’s Hospital, Zhejiang University School of Medicine, National Clinical Research Center for Child Health, Hangzhou, China; ^2^ Department of Cardiac Surgery, The Children’s Hospital, Zhejiang University School of Medicine, National Clinical Research Center for Child Health, Hangzhou, China

**Keywords:** children, septic shock, weighted gene co-expression network analysis, signature gene, immune cells infiltration

In the published article, there was an error.

A correction has been made to **Method and material**, *Identification of DEGs*. This sentence previously stated:

“Using R software’s limma package (19), differentially expressed genes (DEGs) between septic shock cohort and control cohort were analyzed, with the following criterion: p<0.05 and |fold change (FC)| > 1”

The corrected sentence appears below:

“Using R software’s limma package (19), differentially expressed genes (DEGs) between septic shock cohort and control cohort were analyzed, with the following criterion: adjust p value<0.05 and |log fold change (FC)| > 1”

The authors apologize for this error and state that this does not change the scientific conclusions of the article in any way. The original article has been updated.

